# Micronutrient Deficiencies and Related Factors in School-Aged Children in Ethiopia: A Cross-Sectional Study in Libo Kemkem and Fogera Districts, Amhara Regional State

**DOI:** 10.1371/journal.pone.0112858

**Published:** 2014-12-29

**Authors:** Zaida Herrador, Luis Sordo, Endalamaw Gadisa, Antonio Buño, Rubén Gómez-Rioja, Jose Manuel Iturzaeta, Lisset Fernandez de Armas, Agustín Benito, Abraham Aseffa, Javier Moreno, Carmen Cañavate, Estefania Custodio

**Affiliations:** 1 National Centre of Tropical Medicine, Instituto de Salud Carlos III (ISCIII), Madrid, Spain; 2 Tropical Diseases Research Network (RICET in Spanish), Madrid, Spain; 3 National Centre of Epidemiology, ISCIII, Madrid, Spain; 4 Department of Preventive Medicine and Public Health, Faculty of Medicine, Complutense University, Madrid, Spain; 5 Network Biomedical Research Centers, Epidemiology and Public Health (CIBERESP in Spanish), Madrid, Spain; 6 Armauer Hansen Research Institute, Addis Ababa, Ethiopia; 7 University Hospital La Paz, Madrid, Spain; 8 Orense University Hospital, Orense, Spain; 9 National Centre of Microbiology, ISCIII, Madrid, Spain; CINVESTAV-IPN, Mexico

## Abstract

**Introduction:**

The present study describes the distribution of selected micronutrients and anaemia among school-aged children living in Libo Kemkem and Fogera (Amhara State, Ethiopia), assessing differences by socio-demographic characteristics, health status and dietary habits.

**Methods:**

A cross-sectional survey was carried out during May–December 2009. Socio-demographic characteristics, health status and dietary habits were collected. Biomarkers were determined for 764 children. Bivariate and multivariable statistical methods were employed to assess micronutrient deficiencies (MD), anaemia, and their association with different factors.

**Results:**

More than two thirds of the school-aged children (79.5%) had at least one MD and 40.5% had two or more coexisting micronutrient deficiencies. The most prevalent deficiencies were of zinc (12.5%), folate (13.9%), vit A (29.3%) and vit D (49%). Anaemia occurred in 30.9% of the children. Children living in rural areas were more likely to have vit D insufficiency [OR: 5.9 (3.7–9.5)] but less likely to have folate deficiency [OR: 0.2 (0.1–0.4)] and anaemia [OR: 0.58 (0.35–0.97)]. Splenomegaly was positively associated with folate deficiency and anaemia [OR: 2.77 (1.19–6.48) and 4.91 (2.47–9.75)]. Meat and fish consumption were inversely correlated with zinc and ferritin deficiencies [OR: 0.2 (0.1–0.8) and 0.2 (0.1–0.9)], while oil consumption showed a negative association with anaemia and deficiencies of folate and vitamin A [0.58 (0.3–0.9), OR: 0.5 (0.3–0.9) and 0.6 (0.4–0.9)]. Serum ferritin levels were inversely correlated to the presence of anaemia (p<0.005).

**Conclusion:**

There is a high prevalence of vitamin A deficiency and vitamin D insufficiency and a moderate prevalence of zinc and folate deficiencies in school-aged children in this area. The inverse association of anaemia and serum ferritin levels may be due to the presence of infectious diseases in the area. To effectively tackle malnutrition, strategies should target not only isolated micronutrient supplementation but also diet diversification.

## Introduction

Undernutrition encompasses stunting, wasting, and deficiencies of essential vitamins and minerals (collectively referred to as micronutrients) [1]. Micronutrients, which are nutrients that are only needed in minute amounts, play leading roles in the production of enzymes, hormones and other substances. They also help to regulate growth activity, cognitive development and functioning [2], and the activity of the immune and reproductive systems [3]. Besides, micronutrient deficiencies (MD) and especially iron deficiency, is believed to be one of the main underlying causes of anaemia [4].

Micronutrient deficiencies are caused by inadequate dietary intake, increased losses from the body, and/or increased requirements [5]. MD are specially relevant in children since they are in a growth and development phase and have nutritional requirements that vary according to the stage of growth and that are greater and clearly differentiated from those of adults [6]. Recent studies are emphasizing the importance of MD in developing countries [7] and among school-aged children in particular [8]; they are especially vulnerable to inadequate consumption of nutrient-rich foods, dietary taboos, lack of access to health care and inefficient utilization of available micronutrients by cause of infections and parasitic infestations among other reasons [8].

It has become recognized by the nutrition community that micronutrient malnutrition is very widespread, probably one of the main nutritional problems in the world [9] and a major contributor to childhood morbidity and mortality [7], [10], [11]. Micronutrients of known public health importance include the following: zinc, iodine, iron, selenium, copper, vitamins A, E, C, D, B2, B6, B12 and folate [8]. More than 2 billion people in the world today are estimated to be deficient in key vitamins and minerals, mainly vitamin A, iodine and zinc [12]. Particularly in Africa, MD affect millions of people, especially the most vulnerable groups, which are children and pregnant women [13]. Despite the implementation of many micronutrients supplementation programs, only a few countries have undertaken comprehensive surveys on various MD in this continent [14].

In Ethiopia, deficiencies in key vitamins and minerals are placed among the major public health problems [13]. According to the last National Demographic and Health Survey (2011), Ethiopian children aged 6–59 months are dramatically affected by Vitamin A deficiency (VAD) and anaemia, affecting around 61% and 54% respectively [15]. In September 2008, a National Nutrition Strategy, and a National Nutrition Programme (NNP) were launched in the country. This NNP set the need for tackling MD while strengthening nutrition information systems and monitoring and evaluating mechanisms [16].

Although there are some recent studies that have discussed on insufficiencies of some micronutrients in Ethiopian children [8], [17], we are not aware of any recent publication regarding a comprehensive study that includes micronutrients, haemoglobin, food groups intake, as well as health status among children in this area. Therefore, the present study was aimed at describing the distribution of selected micronutrients and the presence of anaemia among school-aged children living in rural and urban areas of Libo Kemkem and Fogera (Amhara Regional State, Ethiopia) and their relationship with diet and health factors.

## Material and Methods

### Location

The study was conducted during May–December 2009 in the districts (*weredas*) of Libo Kemkem and Fogera, located in the Amhara Regional State of Northwestern Ethiopia. According to the 2009 census, the population was 198,374 and 226,595 for Libo Kemkem and Fogera, respectively. Both districts are located in the Tana Zuria Livelihood Zone at an altitude of 1800–2,000 m on average above sea level. Temperatures are relatively high, but rainfall is unusually abundant at 1173 mm per annum as the long-term mean. Agriculture activities are dependent on a single rainy season (from June to September). Maize, barley and millet are the main food crops, while rice, vetch and chickpea are the main cash crops. Livestock holdings in sheep and cattle are relatively modest, but livestock and butter sales make a substantial compliment to the dominant crop sales [18].

### Study design

This cross-sectional study was part of a UBS (Union Bank Switzerland) Optimus Foundation funded project entitled “Visceral Leishmaniasis (VL) and Malnutrition in Amhara State, Ethiopia”, which among its specific objectives aimed to characterize nutritional, immunological, and parasitological aspects of the school-aged children population in the districts of Fogera and Libo Kemkem. Sampling was carried out by multistage cluster survey. Primary sampling units were sub-districts (*kebeles*) with high incidence of VL according to the 2008 register of the Addis Zemen VL Treatment Centre. Secondary sampling units were randomly selected villages (*gotts*) in each of the selected sub-districts. Only one case of active leishmaniasis disease was encountered [19]. Sample size was calculated according to previous estimates of malnutrition for children <5 years old in the area. 889 children aged 4 to 15 years were recruited in Libo Kemkem and Fogera districts, though blood samples were collected only from 764 (85.9%). Study variables did not differ between the complete sample and those from whom blood samples were obtained at the 0.05 probability level (S1 Table). Other methodological aspects have been published elsewhere [19], [20].

### Data collection

A uniform pre-piloted questionnaire translated into Amharic was administered to the caretaker/head of household of the participant children by trained medical personnel (nurses and health officers). The questionnaire comprised the following parts: demographic characteristics, health status and diet factors. The following information regarding health status was collected during the interview: presence of splenomegaly, fever in the last 15 days and weight loss. Dietary data were collected through a 24-hour diet recall.

A blood sample was taken via venipuncture from the selected children in order to determine serum levels of micronutrients.

### Determination of levels of micronutrients and anaemia

Around 7 milliliters (ml) of venous blood were collected in a serum tube and 1 ml in a tube containing Na2-EDTA (Sigma, St. Louis, MO), then transported to the Regional Health Laboratory of Bahir Dar in cool boxes at a temperature of 4–8°C. Within 24 hours from the sample recollection a lab technician centrifuged and separated the serum of the blood contained in the serum tube, and stored it at −20°C and protected from light, the conditions in which the samples were shipped to the Clinical Laboratory of the University Hospital La Paz in Madrid (Spain) by using dry ice. Once there, they were stored at −20°C and protected from the light until the analysis on micronutrients were performed. The content of the EDTA tube was used for the complete cell blood count (haemogram) the day after the blood had been collected.

Micronutrients determinations were carried out according to availability of serum volume and following a pre-defined prioritization list. The following techniques were used: zinc and copper were determined by atomic absorption spectroscopy (3110 atomic absorption spectrometer Perkin Elmer). Ferritin, folate and vitamin B12 were determined by electrochemiluminescence immunoassay (Elecsys E170, Roche Diagnostics), vitamin D by chemiluminescent immunoassay (Liaisson, DiaSorin), and vitamins A and C by high-performance liquid chromatography (HPLC) (1200 series, Agilent Technologies). The mean interday coefficient of variation (CV) for all the parameters and information related to the external quality control schemes used are presented in S2 Table.

Cut-off values for micronutrients deficiencies and anaemia were defined after a review of the relevant literature. These values and their units are summarized in Table 1.

**Table 1:** Cut-off values for selected micronutrients deficiency and anaemia.

Micronutrient	Cut-off values	Reference
**Zinc**	<65 µg/dL (children<10 years); <70 µg/dL (children≥10 years)	(25)
**Copper**	<0.90 µg/L	(26)
**Serum Folate**	<10 nmol/L	(27)
**Vit B12**	<150 pmol/L	(27)
**Vit A**	<0.70 µmol/L	(28)
**Vit D** [1]	<75 nmol/l	(29)
**Vit C**	<11.4 µmol/L	(30)
**Ferritine**	<15 µg/L for males; <12 µg/L for females	(12)
**HGB** [2]	<118 g/l if (children<5 years); <123 g/l (children 5–11 years); <128 g/l (children ≥ 12 years)	(31)
**MCV**	<81 fl if <4.9 years; <82 fl if <7.9 years; <84 fl if <11.9 years;	(32)
	For children>12 years: <85 fl for males; <86 fl for females	

[1] Vitamin D insufficiency was defined as serum concentrations below 75 nmol/l according to an expert consensus.

[2] Adjusted by altitude and age according to WHO standards.

### Statistical analysis

SPSS version 18.0 (SPSS Inc., Chicago, IL) was used to enter and analyze data. Based on the FAO/FANTA Household Dietary Diversity Questionnaire and Guidelines [21], the dietary data collected through the 24-hour diet recall were computed into 9 food groups: cereals, roots and tubers; vitamin-A-rich fruits and vegetables; other fruit; other vegetables; legumes and nuts; meat, poultry and fish; fats and oils; dairy; and eggs. The Dietary Diversity Score (DDS) was calculated by summing the number of unique food groups. This index is based on a simple count of food groups consumed in the previous day to the survey. Accordingly, the level of diet diversity was computed out of the score of 9.

To understand the descriptive epidemiology of micronutrients deficiency, we computed plasma values into lower quartile (below 25th percentile), middle quartile (≥25th percentile and ≤75th percentile), and upper quartile (baseline plasma values >75th percentile). Applying selected cutoff values for MD, we estimated the global percentages.

Frequencies, means and standard deviations (SD) were used to summarize the data. Bivariate analyses for MD of statistical relevance (prevalence of deficiency >10%) and their related factors were performed by student's t-test and χ2 tests for continuous and categorical variables, respectively.

Multivariable logistic regression models for micronutrient deficiencies and anaemia were obtained by using a manual backward stepwise procedure. Age, sex and setting, considered biologically and statistically relevant, and all variables associated with each of the outcomes at the p<0.10 level in the bivariate analysis were included. To assess anaemia, ferritin, vitamin B12 and folate serum levels were also included in the analysis. The major assumptions of logistic regression analysis (absence of multicollinearity and interaction among independent variables) were checked to be satisfied. The goodness of fit was assessed using Hosmer-Lemeshow statistic. The adjusted odds ratio (OR) and 95% confidence interval (95% CI) were computed. P-values less than or equal to 0.05 were considered statistically significant.

### Ethical considerations

The study was approved by the ethical review boards of Instituto de Salud Carlos III, the Armauer Hansen Research Institute (AHRI)/All Leprosy Rehabilitation & Training Center (ALERT) and the Ethiopian National Ethical Review Committee. Support letters were obtained from the Amhara Regional State's and district's health bureaus. All parents/guardians gave written informed consent before enrollment of their children in the study.

## Results and Discussion

### 1. Socio demographic characteristics and dietary habits

The study included a total of 764 children aged 4 to 15 years out of which 49.7% were females and 21.2% were living in urban areas. Regarding health status related variables, 36% of the recruited children had had fever in the 15 days prior to the survey while almost 21% had had weight loss (Table 2).

**Table 2:** Distribution of selected characteristics of school-aged children, Libo Kemkem and Fogera, Ethiopia, May-December 2009 (n = 764).

CHARACTERISTICS	%/Mean(sd)
	
**DEMOGRAPHIC**	
% Female	49.74
Mean age (sd)	9.03 (3.18)
% living in urban communities	21.2
**HEALTH STATUS**	
% had splenomegaly	6.83
% had fever in the last 15 days	36.04
% had weight loss in the last 15 days	20.71
**DIET HABITS** (the day before the survey)	
% Children consumed basic staples	99.87
% Children consumed VitA rich fruits and vegetables	2.49
% Children consumed other fruits	0.39
% Children consumed other vegetables	12.43
% Children consumed legumes and pulses	85.47
% Children consumed meat or fish	16.88
% Children consumed oil	79.71
% Children consumed dairy	14.01
% Children consumed eggs	1.05
Mean food groups (sd)	3.13 (0.72)

According to the 24-h recall interviews, the mean DDS in the referenced period was 3.13 (SD = 0.72), with adequate dietary diversity (four or more food groups consumed the previous day) attained only by 22.9% of the study population. The diet contained mostly basic staples, legumes, pulses and oil. Less than 17% and 15% of the interviewees reported intake of meat/fish or dairy products in the previous day, respectively, and no child consumed more than six food groups in the previous 24 hours (S3 Table). Overall, the Ethiopian diet is mainly composed of cereals (maize, sorghum and *teff*), tubers and root crops, pulses and oil seeds. Despite a large local livestock population, the food supply of animal products has been previously reported to be very limited, especially in rural areas [22].

Our result of low DDS is consistent with previous studies carried out in Ethiopia; in a review which compared DDS results from 11 developing countries, the lowest mean DDS was found in Mali, followed by Ethiopia and Malawi [23]. Theoretical and empirical evidence suggests that DDS is an effective food and nutrition security indicator because it captures consumption of both macro and micronutrients and also reflects food diversification [24]. Other studies on diet quality in Ethiopia indicate that there is a high prevalence of food insecurity with important spatial characteristics; in the Amhara regional state a significant proportion (45%) of rural households are described as food insecure [25]. Despite international agreement on the important role of the diet diversity (DD) for infants and young children [2], there are currently no specific recommendations regarding the optimal number of foods or food groups that a child should consume daily at different ages [26]. There is, however, a consensus that higher DDS is desirable and that a larger number of foods or food groups can help meet daily requirements for a variety of nutrients [27].

### 2. Micronutrients determinations: serum levels and deficiencies

In this study, 53.5% of the school-aged children had at least one MD and 21.4% had two or more coexisting micronutrient deficiencies. The prevalence of MD varied widely by micronutrient: from a minimum of 1.3% for vitamin B12 to a maximum of 49% for vitamin D insufficiency (Table 3).

**Table 3:** Levels of serum micronutrients and anaemia among school-aged children in Libo Kemkem and Fogera, Ethiopia, May-December 2009.

Micronutrient	Unit	Mean (sd)	Median	Quartiles			Deficiency*	
				Q25	Q50	Q75	n	%
Zinc (n = 618)	µg/dL	86.89 (19.63)	85.00	74.00	85.00	97.00	77	12.5
Copper (n = 660)	µg/dL	135.60 (28.20)	133.00	118.00	133.00	150.00	22	3.3
Folate (n = 534)	nmol/L	17.11 (7.35)	15.52	11.78	15.52	21.30	74	13.9
Vit B12 (n = 532)	pmol/L	405.36 (166.43)	380.44	287.82	380.44	482.65	7	1.3
Vit A (n = 663)	µmol/L	0.92 (0.47)	0.84	0.63	0.84	1.09	194	29.3
Vit D (n = 627)	nmol/L	80.10 (26.42)	77.38	62.40	77.38	94.85	307	49.0
Vit C (n = 322)	µmol/L	31.98 (12.83)	31.00	23.20	31.00	39.63	13	4.0
Ferritin (n = 533)	µg/L	64.39 (51.75)	49.00	33.00	49.00	76.50	18	3,4
Haemoglobine (n = 764)	g/L	130.04 (17.05)	13.10	122.00	131.00	139.75	236	30.9
MCV (n = 236)**	fL	82.86 (5.57)	83.00	80.00	83.00	86.10	154	65.3

sd: standar deviation; * Cut off values defined in Table 1; ** Only from those children with anaemia

#### 2.1. Low prevalence deficiencies: copper, ferritin and vitamins B12 and C

The mean serum level of copper in our study population was 135.60±28.20 µg/dl. In a previous research carried at Gondar town, in Amhara Regional State, Ethiopia, Amare et al. found a mean value of 191.30±50.17 µg/dl in 100 urban school-aged children [28], figures which are higher than our findings. This difference may be partially accounted for by the lack of rural communities in their sample, taking into account that rural-urban disparities for this micronutrient have been previously reported in the literature [29]. Furthermore, the children from the Amare et al. study are children attending school while the children from our study are school-aged children that do not necessarily attend school, and may have worse socio environmental living conditions. Another research, also placed in the Gondar zone (Ethiopia) and targeting non-pregnant women [30], found a lower mean level of copper (146.8±49.4, 36), which was more alike our result, although the study group was different and results are therefore not fully comparable.

Ferritin mean serum level was 64.39±51.75 µg/L. The standard deviation for this mean value was quite large despite that no outlier value was identified. It is common to find this kind of wide ranges for this parameter in the literature; Adebara *et al*. carried out a research on a similar age group (5–12 years old school children) in an urban city from Nigeria, and found a mean serum ferritin level of 77.6±32.6 µg/L, with a prevalence of ferritin deficiency (FED) of 3.7%; results that are in consonance with ours [31].

Mean level of vitamin B_12_ was 405.36 pmol/L (±166.43). The high standard deviation indicates that our data is spread out over a large range of values. This is in concordance with national level survey data from different countries [32]. In the literature, the measures of central tendency of serum concentrations of vitamin B12 ranged from 100 pmol/L in men in a local Cuban survey [33] to 779 pmol/L in non-pregnant women in a local survey in the Republic of Korea [34], with wide diversity of targeted groups, locations and lab techniques among researches. Consequently, the comparison of this particular deficiency is limited [32].

Regarding Vitamin C, the mean level in the children of our study was 32±12.8 µmol. Although we obtained a lower mean serum level than the one reported by the United States National Health and Nutrition Examination Survey (NHANES) for children >6 years (51.4±3 µmol), which was our reference study for this particular MD, the deficiency rate was higher in the American study (7.1% vs 4%)[35]. This fact highlights the need of computing both central tendency figures and deficiency rates to better understand the distribution of MD in study populations.

#### 2.2. High prevalence deficiencies: zinc, folate, vitamin A and vitamin D

##### 2.2.1 Zinc deficiency

The mean serum level in our population was 86.89±19.63 µg/dl. Although this level was similar to the one found by Amare *et al.* (86.40±42.40 µg/dl) in school-aged children in a nearby area [17], our deficiency rate was considerably lower (12.5% vs 47%). This can be due to the different cut-off values used in both studies; while we used the one proposed by the International Zinc Nutrition Consultative Group (IZiNCG) showed in Table 1 [36], Amare used Sauberlich's “Review on Laboratory Methods for the Assessment of Nutritional Status”, that is <75 µg/dl for all ages [37]. This remarks the importance of having internationally agreed cut-off values for MD to better explore and explain data differences.

We found that younger children presented a significantly lower prevalence of zinc deficiency (ZD) than older children (p = 0.014); although no differences were found by sex (see Table 4). This is consistent with results reported by the IZiNCG, stating that 21.1% of the Ethiopian population was at risk of inadequate dietary zinc intake [38].

**Table 4:** Risk factors for selected micronutrient deficiencies in school-aged children in Libo Kemkem and Fogera, Ethiopia, May-December 2009.

Deficiency	*P* value	*Adjusted OR (95% CI)*	*Goodness of Fit*
**Zinc deficiency (12%)**			
Sex (male)	0.485	1.19 (0.73–1.97)	R^2^ Nagelkerke = 0.07; Hosmer-Lemeshow (sig) = 0.129
Age (years)	0.014	1.10 (1.02–1.19)	
Setting (rural)	0.079	2.33 (0.91–5.99)	
Consumption of legumes and pulses*	0.035	0.37 (0.15–0.93)	
Consumption of meat*	0.025	0.22 (0.06–0.83)	
Diet diversity score (DDS)*	0.067	1.42 (0.98–2.05)	
**Folate deficiency (13.9%)**			
Sex (male)	0.807	0.94 (0.56–1.58)	R^2^ Nagelkerke = 0.12; Hosmer-Lemeshow (sig) = 0.235
Age (years)	0.365	1.04 (0.96–1.13)	
Setting (rural)	0.000	0.20 (0.10–0.43)	
Had splenomegaly	0.019	2.77 (1.19–6.48)	
Fever in the last 15 days	0.008	0.42 (0.22–0.80)	
Consumption of meat*	0.067	0.43 (0.17–1.06)	
Consumption of oil*	0.036	0.53 (0.29–0.96)	
**Vit A deficiency (29.3%)**			
Sex (male)	0.285	1.21 (0.85–1.71)	R^2^ Nagelkerke = 0.06; Hosmer-Lemeshow (sig) = 0.693
Age (years)	0.286	0.97 (0.92–1.03)	
Setting (rural)	0.162	1.49 (0.85–2.62)	
Fever in the last 15 days	0.025	1.51 (1.05–2.17)	
Consumption of meat*	0.063	0.51 (0.25–1.04)	
Consumption of oil*	0.030	0.57 (0.35–0.95)	
Diet diversity score (DDS)*	0.068	1.32 (0.98–1.79)	
**Vit D deficiency (49%)**			
Sex (male)	0.000	1.85 (1.32–2.60)	R^2^ Nagelkerke = 0.18; Hosmer-Lemeshow (sig) = 0.291
Age (years)	0.001	1.09 (1.04–1.16)	
Setting (rural)	0.000	5.96 (3.74–9.49)	
Consumption of other vegetables*	0.061	0.61 (0.36–1.02)	

*The day before the survey

The probability of ZD was 0.22 (95 CI: 0.06–0.83) and 0.37 (95% CI: 0.15–0.93) times lower among the children who had consumed meat/fish or legumes/pulses the day before, respectively. Foods rich in zinc and potassium belong to the meat, the legume and the grain food groups, although some unrefined cereals and legumes may contain a high content of phytate, a substance that significantly inhibits the body's absorption of this micronutrient [39]. Similar results were obtained in relation to meat consumption and zinc status in pregnant women in another research carried in a southern area of Ethiopia [40].

Up to date, research on zinc in Ethiopia has been mostly focused on pregnant women [30], [41], [42]. Further research in the study area should be conducted to assess the determinants of ZD in different age and sex groups.

##### 2.2.2. Folate deficiency

Mean level of serum folate in our study population was 17.11±7.35 nmol/L and 13.9% of the school-aged children surveyed were folate deficient. Folate Deficiency (FD) has been described as a frequent complication of protein-energy malnutrition in both West and Southern Africa [43]. A recent review by WHO recognized the lack of universally accepted reference levels to define FD as a public health problem and expressed the need for consensus on this issue [44]. We used here the cut-off value proposed in that review. As it occurs with zinc, most research in Ethiopia regarding folate status has been carried out only in pregnant women [42], [45] and it seems not to be a current priority intervention for the health authorities [46]. Therefore, although we may not be able to compare our results with similar age groups, we do consider these data of interest as a starting point for further research on this topic.

In our study, FD was significantly more common among children living in urban settings rather than in rural ones (p<0.001; Table 4). This result may be associated with the higher prevalence of anaemia found in the urban communities. FD also seems to be inversely related with vitamin B12 deficiency [44]; a large local survey in the district of Embu (Kenya) revealed that 40% of school-aged children had plasma vitamin B12 deficiency, however only 1% had serum folate deficiency [47]. This inverse association also seems to exist in our population, though further research involving other relevant biological factors is needed to better explore this hypothesis.

Splenomegaly was significantly and positively associated with FD (p<0.05) While having an episode of fever in 15 days prior to the survey was inversely associated with it (p = 0.008). Malaria or other infections may be the intermediates of these associations, although we did not collect any specific data on these infections and thus we were not able to adjust by any of them.

##### 2.2.3. Vitamin A deficiency

The serum mean value for vitamin A was 0.92±0.47 µmol/L. This mean value is higher than the one reported in 6–9 years old children from Wukro [48], in Northern Ethiopia (0.69 µmol/L ±0.31). The 29.3% of our study population was vitamin A deficient (VAD). In a national vitamin A survey targeting children aged 6 to 71 months and their respective mothers, 33% of population from Amhara was VAD [49]. Since both studies, the national survey and the research carried in Wukro, targeted different age groups, comparisons of data on prevalence would be inappropriate. Moreover, the present study was conducted in two single sites in Ethiopia thus we cannot extrapolate our results to other areas of Ethiopia. VAD is widely reported as a health problem in developing countries, affecting mainly children and women at child bearing age [10]. In Ethiopia, VAD was identified as a public health concern already in 1958 [50]. Subsequent surveys revealed low dietary vitamin A intake except in the southern region [46]. There are many possible causes for this nutritional deficiency, ranging from low vitamin A-rich foods intake, problems with the absorption, conversion or utilization of vitamin A or the suffering of frequent infections or diseases [46]. In our study population, school-aged children who had fever in 15 days prior to the survey were 1.51 times (95% CI: 1.05–2.17; Table 4) more likely to have VAD. This may reflect an association of VAD with a past infection [49] although the association with lower intake of vitamin A cannot be discarded and should also be heeded.

Children who consumed oil in the previous 24 hours were significantly less likely to have VAD (p<0.05). Vitamin A is a fat-soluble vitamin, which means that consumption of oils or fats are necessary for its absorption into the body [21]. Therefore, if a diet is lacking oils and fats, vitamin A is not well absorbed and utilized. Moreover, palm oil, which is one of the most widely consumed oil in Ethiopia, is a rich source of carotenoids [51].

Applying WHO cut-off points for interpretation in terms of public health significance, VAD is a severe public health problem in the study area (≥20%) [52]. According to FAO country profile report, Vitamin A supplementation coverage remains limited, especially in rural areas in Amhara and Tigray regions [22]. Our results support the need to reevaluate the practice of targeting vitamin A supplementation programs on school-aged children in this area.

##### 2.2.4. Vitamin D insufficiency

Mean value of 25-hydroxyvitamin D (25(OH) D) in serum was 80.10±26.42 nmol/L. Vitamin D studies in Africa have recently been reviewed by Prentice A *et al.* [53], although for Ethiopia they only provided vitamin D serum values for adult men and non-pregnant women (23.5 nmol/l) and, therefore we cannot compared our results.

In the past decade, considerable discussion has taken place regarding the definition of vitamin D sufficiency. A recent expert consensus considered optimal status to be at serum concentrations of 25(OH) D>75 nmol/l [54]; thus values under this cut-off point are categorized as vitamin D insufficiency but not deficiency. Considering so, the prevalence of insufficiency (VDI) in our study population was 49%.

Lower age and male sex were significantly associated to higher prevalence of VDI (p<0.001; Table 4). In the scientific literature, it is commonly agreed that people at extremes of age (young children and older individuals) are more vulnerable to VDI [55], whereas sex differences are generally found in the opposite direction to our results; VDI being more prevalent among women [56]. Risk factors for VDI which have been previously described in developing countries include winter season, dark skin pigmentation, malnutrition, lack of sun exposure and a covered clothing style [56]. In a case-control study targeting Ethiopian children living in Addis Ababa, Lulseged *et al*. found that poor exposure to radiant energy was the main cause of VDI [57]. Since the level of vitamin D can be modified by seasonal variations and age [58], longitudinal studies are needed to assess this insufficiency.

School-aged children living in rural areas were 5.95 (95% CI: 3.74–9.49) times more likely to suffer VDI than those living in urban communities. Although we did not identify any diet factor associated with the prevalence of VDI, we are aware of significant differences in dietary habits among selected rural and urban communities [59], which may at least partially explained these differences. Furthermore, the higher prevalence of VDI among rural children as opposed to urban ones had been previously reported in one study conducted in Ethiopia, in which the difference was attributed to traditional beliefs and practices affecting the frequency of exposure to sunlight [60].This has also been described for other Africa countries lying within the tropics and subtropics [61].

### 3. Anaemia

Hb mean serum level was 130.04±17.05 g/L. This finding is consistent with previous studies in school children in other Ethiopian regions [62], [63]. Around 31% of the school-aged children in our study had anaemia. This result is markedly higher than the 9.8% figure for school children from the Ethiopian National Survey of Health and Nutrition [64], however it is important to highlight that the cited figure was a mean value of the 11 Ethiopian regions, and may be masking important inter regional disparities. Furthermore, our result is consistent with the 35,2% anemia prevalence for children aged 6 to 59 months living in Amhara region reported by the Ethiopian Demographic and Health Survey (DHS) in 2011 [15]. However, this heterogeneity should be noted: regional and environmental factors may impact anaemia prevalence in this context, and therefore should be further investigated on future studies.

The mean corpuscular volume (MCV) in the study population was 82.85 fL (SD: 5.57), which is lower than the normal values proposed by WHO [65]. A low MCV, suggesting microcytic anaemia, occurs in several childhood disorders including iron deficiency anemia, beta-thalassemia trait, lead poisoning, anemia from chronic illness, and rarely, in sideroblastic anemia [66].

The prevalence of anaemia was significantly higher in urban (39.5%) than in rural (28.6%) settings (p<0.005, Table 5). This result is discordant with common findings which suggest the existence of worse health indicators in rural settings [13]. Implications of urban growth, bad eating habits or unequal implementation of supplementary nutritional programs may explain these differences [67], although local investigations are needed to better assess these disparities. The odd for having low Hb serum level was around 5 times more likely in those children who presented splenomegaly. Similar results were found by Haidar *et al.* in nine regions of Ethiopia [68]. The etiology of anemia is one of multiple and interacting causes; common causes of anemia include nutritional deficiencies of iron, vitamin B12 and folic acid, but also malaria, intestinal parasites and some chronic disorders secondary to AIDS and tuberculosis [43]. In our research, splenomegaly may represent a current parasitic infection, thus other health indicators are desirable to fully explain this association.

**Table 5:** Risk factors for anaemia among school-aged children in Libo Kemkem and Fogera, Ethiopia, May-December 2009 by outcome.

Deficiency	*P* value	Adjusted OR (95%CI)	Model's goodness of fit
**Anaemia (30.9%)**			
Sex (male)	0.465	1.16 (0.78–1.73)	R^2^ Nagelkerke = 0.131; Hosmer-Lemeshow (sig) = 0.671
Age (years)	0.123	0.95 (0.89–1.01)	
Setting (rural)	0.037	0.58 (0.35–0.97)	
Had splenomegaly	0.000	4.91 (2.47–9.75)	
Consumption of oil*	0.032	0.58 (0.35–0.95)	
Ferritin serum level (µg/L)	0.000	1.01 (1.00–1.01)	

*The day before the survey.

The consumption of oil was inversely associated with anaemia [OR: 0.58 (CI95%: 0.35–0.95)]. The development of anaemia in children may be caused by poor diet. In Ethiopia, oils consumed at household level are mainly made out of palm seeds and soybeans [69]. A case-control study carried out in India found that the proportion of women having anemia was significantly lower in the palm oil-supplemented group [51]. The benefits of the consumption of these types of oil may be mediated through other key elements, considering that palm oil is a rich source of micronutrients like vitamin A.

In the logistic regression analysis, no significant association between anaemia and vitamin B12 and/or folate serum levels was found, while serum ferritin was positively correlated to the presence of anaemia (p<0.005). According to our results, iron stores, measured by serum ferritin levels were adequate (with only 3.4% of the children having them exhausted) (Table 3), however, it is important to highlight that serum ferritin levels are elevated by the presence of infection and/or inflammation processes and, therefore, may not be a good proxy for iron stores in populations with high prevalence of parasitic infections or other infectious diseases. On the other hand, infectious diseases such as malaria, may lower haemoglobin concentrations and lead to anaemia [11], [70], resulting in this apparent contradiction of the inverse association between ferritin and haemoglobin levels. In order to contrast this hypothesis studies that take concurrent measurements of normal C-reactive protein and/or α1-acid-glycoprotein (AGP) are needed.

### 4. Limitations

The present study was conducted in two single sites in Ethiopia. Thus, the findings may not be generalizable to a larger population. Additionally, the cross-sectional nature of the data does not allow examining causality in the relationship between micronutrient deficiencies and associated factors.

In Ethiopia, seasonal under nutrition has been previously described as highly unpredictable, with considerable variations in the impact of seasonal stress within localities and even within households [71]. In consequence, we decided not to assess the role of seasonality in MD prevalence. However, we are aware that periodical and longitudinal measurements may be desirable to test this hypothesis in this specific study area.

Pre-analytical bias due to the complex field logistics and the samples transport might have occurred. To reduce these potential biases, detailed guidelines on samples collection, preservation and transport were prepared and piloted prior to the field work. All these protocols were also tested by internal control before and during the field work.

Vitamin C was not collected in a solution to preserve degradation and, therefore, results of this micronutrient may be taken with caution. However, due to the scarcity of studies analyzing vitamin C serum level in this population we considered of interest to include it in the results despite this limitation.

Micronutrient deficiencies and infectious diseases often coexist and exhibit complex interactions. Several micronutrients have immunomodulating functions and thus influence the susceptibility of a host to infectious diseases and the course and outcome of such diseases [72], [73]. Moreover, changes in levels of acute phase proteins such as C - reactive protein (CRP) are associated with increased plasma levels of some micronutrients, such as ferritin, and decrease of others, such as retinol. Therefore, an important limitation of the present study is the lack of more detailed information on infectious status, from a qualitative and quantitative approach (i.e. complete assessment of self-perceived morbidity and measurement of CRP or other biological markers). Nonetheless, our study area is known to be a low endemic area for some endemic diseases such as malaria and leishmaniasis [19], [74].

Micronutrients serum levels may also be artificially affected by the time of day of blood sample collection, the fasting status of the study subjects and/or the seasonality of certain conditions such as the incidence of endemic infectious diseases and the food availability, as it occurs with zinc [36]. Therefore, consecutives and prospective measures are needed to better explore these associations over time. Such data may provide useful information to explain MD in this population.

The reader is also alerted to limitations inherent to the nature of this study, namely its descriptive nature and the lack of methodological standardization of some micronutrient measurements and cut-off values at international level. The lack of agreement on cut off values for different targeted population occur either because of the absence of a suitable biomarker of deficiency or simply because, to date, little investigation has taken place [44], [75]. These calls for the need to undertake further investigations not only to substantiate the data obtained in the present study but also to check new hypothesis which may have emerged.

## Conclusions

Our findings reveal a high prevalence of vitamin A deficiency and vitamin D insufficiency (29.3% and 49%, respectively) while moderate prevalence of zinc and folate deficiencies (12.5% and 13.9%, respectively) in school-aged children in Libo Kemkem and Fogera. The magnitude of anaemia determined in this study (30.9%) is considered as a moderate public health problem according to WHO standards [65]. Up to date, only few countries have conducted surveys on micronutrients status at the national or subnational level, and most of them are focused on limited number of micronutrients [47], [48], [68]. Given the paucity of previous data, our results represent a starting point for future research.

To effectively tackle micronutrients deficiencies and anaemia in school-aged children, nutritional programs should be oriented to the local needs. Zinc supplementation trials have clearly demonstrated the positive benefits of improved zinc status in children, including reductions in the incidence of various infectious diseases [26], [36]. There is also enough evidence of the benefits of vitamins A and D supplementation programs in similar contexts [10], [26], [76]. Targeting anaemia is a complex challenge in developing countries, where prevalence of malnutrition and infectious diseases is high, although until date, iron supplementation and nutrition education are the only interventions that seem to be effective against IDA [26].

Micronutrient-deficiency control programs have been greatly extended in most African countries [14], although most interventions have been focused on single micronutrients and often lack effectiveness [77]. Diet diversification strategies are far less common, despite of dietary diversity being universally recognized as a key component of nutrition programs [9]. Therefore, major efforts are needed to close critical knowledge gaps, to test and scale-up improved and affordable interventions, and to disseminate evidence to decision makers and stakeholders to fully respond to this key public health problem. This will help to rapidly and sustainably improve the diet diversity and the nutritional status in the study setting, which would have a positive impact on economic growth and development.

Findings documented by this study would assist in planning and undertaking regional policy initiatives for diet diversification strategies to streamline recommendations. Monitoring activities should also follow these interventions to better assess their impact on micronutrients deficiencies and anaemia. This will enable the right interventions to be chosen and then, once programs are in place, to have the right indicators to follow-up these activities [11].

## Supporting Information

S1 Table Distribution of selected characteristics of study children (sample and sub-sample with serum collection), Libo kemkem and Fogera, Ethiopia, May-December 2009.
(DOCX)

S2 Table Performance characteristics of the biochemical parameters measured.
(DOCX)

S3 Table Percent consumption of different food groups by DDS for school-aged children in Libo kemkem and Fogera, Ethiopia, May-December 2009.
(DOCX)
