# Supplementary material for: Micronutrient Deficiencies and Related Factors in School-Aged Children in Ethiopia: A Cross-Sectional Study in Libo Kemkem and Fogera Districts, Amhara Regional State
Source: PLoS One. 2014 Dec 29;9(12):e112858. doi: 10.1371/journal.pone.0112858 (PMC4278675; doi:10.1371/journal.pone.0112858)
Supplement: S1 Table — Distribution of selected characteristics of study children (sample and sub-sample with serum collection), Libo kemkem and Fogera, Ethiopia, May-December 2009. (DOCX) [file pone.0112858.s001.docx]

| **Table S1. Distribution of selected characteristics of study children (sample and sub-sample with serum collection), Libo kemkem and Fogera, Ethiopia, May-December 2009** | | | |
| --- | --- | --- | --- |
| **CHARACTERISTICS** | **Sample** | **Sub-sample** | **p value** |
|  | **n=889** | **n=764** |  |
| **DEMOGRAPHIC** | | | |
| % Female | 48.03 | 49.73 | p>0.005 |
| Mean age (sd) | 9.05 (3.15) | 9.03 (3.18) | p>0.005 |
| % Living in urban communities | 20.02 | 21.20 | p>0.005 |
| **HEALTH STATUS** | | | |
| % Had splenomegaly | 6.43 | 6.83 | p>0.005 |
| % Fever in the last 15 days | 35.36 | 36.04 | p>0.005 |
| % Had weight loss? | 19.93 | 20.71 | p>0.005 |
| **DIET HABITS (the day before the survey)** | | | |
| %Child consumed any basic staples | 99.77 | 99.87 | p>0.005 |
| %Child consumed any VitA rich fruits and vegetables | 2.81 | 2.49 | p>0.005 |
| %Child consumed any other fruits | 0.34 | 0.39 | p>0.005 |
| %Child consumed any other vegetables | 11.81 | 12.43 | p>0.005 |
| %Child consumed any legumes and pulses | 85.83 | 85.47 | p>0.005 |
| %Child consumed any meat or fish | 16.31 | 16.88 | p>0.005 |
| %Child consumed any oil | 81.10 | 79.71 | p>0.005 |
| %Child consumed any dairy | 13.39 | 14.01 | p>0.005 |
| %Child consumed any eggs | 1.12 | 1.05 | p>0.005 |
| Mean food groups (sd) | 3.13 (0.71) | 3.13 (0.72) | p>0.005 |
