# Supplementary material for: Micronutrient Deficiencies and Related Factors in School-Aged Children in Ethiopia: A Cross-Sectional Study in Libo Kemkem and Fogera Districts, Amhara Regional State
Source: PLoS One. 2014 Dec 29;9(12):e112858. doi: 10.1371/journal.pone.0112858 (PMC4278675; doi:10.1371/journal.pone.0112858)
Supplement: S2 Table — Performance characteristics of the biochemical parameters measured. (DOCX) [file pone.0112858.s002.docx]

| **Table S2: Performance characteristics of the biochemical parameters measured.** | | | | |
| --- | --- | --- | --- | --- |
| **Parameter** | **Units** | **Level** | **Coefficient of Variation (%)** | **External Quality Control** |
| **Vit A** | **µg/dL** | 0,19 | 10,4 | No |
|  |  | 0,8 | 10,1 |  |
| **Vit C** | **µmol/L** | 12,3 | 12,5 | No |
|  |  | 25,6 | 9,6 |  |
| **Copper** | **µg/dL** | 64 | 3,7 | SEQC*/SFBC** |
|  |  | 94 | 3,6 |  |
| **Zinc** | **µg/dL** | 118 | 8,1 | SEQC/SFBC |
|  |  | 208 | 8,2 |  |
| **Ferritine** | **µg/L** | 22 | 5,7 | SEQC |
|  |  | 130 | 4,1 |  |
| **Folate** | **µg/dL** | 3,21 | 7,7 | SEQC |
|  |  | 6,71 | 10,7 |  |
| **Vit B12** | **pg/mL** | 213 | 9,4 | SEQC |
|  |  | 454 | 6,9 |  |
| **Vit D** | **ng/mL** | 42 | 10,3 | UK-NEQAS*** |
|  |  | 99 | 6,6 |  |
| *SEQC: Sociedad Española de Bioquímica Clínica y Patología Molecular | | | | |
| **SFBC: Societe Francaise de Biologie Clinique | | | |  |
| ***UK-NEQAS: United Kingdom National External Quality Assessment Service | | | | |
