# Supplementary material for: Micronutrient Deficiencies and Related Factors in School-Aged Children in Ethiopia: A Cross-Sectional Study in Libo Kemkem and Fogera Districts, Amhara Regional State
Source: PLoS One. 2014 Dec 29;9(12):e112858. doi: 10.1371/journal.pone.0112858 (PMC4278675; doi:10.1371/journal.pone.0112858)
Supplement: S3 Table — Percent consumption of different food groups by DDS for school-aged children in Libo kemkem and Fogera, Ethiopia, May-December 2009. (DOCX) [file pone.0112858.s003.docx]

| **Table S3. Percent consumption of different food groups by DDS for school-aged children in Libo kemkem and Fogera, Ethiopia, May-December 2009** | | | | | | | | | |
| --- | --- | --- | --- | --- | --- | --- | --- | --- | --- |
| DDS | Basic staples | VitA rich fruits and vegetables | Other fruits | Other vegetables | Legumes and pulses | Meat/Fish | Oil | Dairy | Eggs |
| **1** | 100.0 | 0.0 | 0.0 | 0.0 | 0.0 | 0.0 | 0.0 | 0.0 | 0.0 |
| **2** | 100.0 | 0.0 | 0.0 | 0.81 | 77.24 | 6.50 | 7.32 | 6.50 | 1.63 |
| **3** | 100.0 | 0.18 | 0.0 | 3.92 | 86.45 | 11.59 | 91.80 | 6.06 | 0.0 |
| **4** | 100.0 | 3.77 | 0.0 | 32.08 | 90.57 | 33.33 | 97.48 | 40.25 | 2.52 |
| **5** | 100.0 | 41.46 | 7.32 | 73.17 | 92.68 | 43.90 | 100.0 | 31.71 | 9.76 |
| **6** | 100.0 | 100.0 | 0.0 | 100.0 | 100.0 | 100.0 | 100.0 | 0.0 | 0.0 |
| **7** | ─ | ─ | ─ | ─ | ─ | ─ | ─ | ─ | ─ |
| **8** | ─ | ─ | ─ | ─ | ─ | ─ | ─ | ─ | ─ |
| **9** | ─ | ─ | ─ | ─ | ─ | ─ | ─ | ─ | ─ |
